# Comorbidities and Lack of Blood Transfusion May Negatively Affect Maternal Outcomes of Women with Obstetric Hemorrhage Treated with NASG

**DOI:** 10.1371/journal.pone.0070446

**Published:** 2013-08-08

**Authors:** Alison El Ayadi, Sarah Raifman, Farouk Jega, Elizabeth Butrick, Yemisi Ojo, Stacie Geller, Suellen Miller

**Affiliations:** 1 Department of Obstetrics, Gynecology & Reproductive Sciences, University of California San Francisco, San Francisco, California, United States of America; 2 Department of Global Health and Population, Harvard School of Public Health, Boston, Massachusetts, United States of America; 3 Pathfinder International, Abuja, Nigeria; 4 Department of Obstetrics & Gynecology, College of Medicine, University of Illinois Chicago, Chicago, Illinois, United States of America; University of Vermont College of Medicine, United States of America

## Abstract

The Non-Pneumatic Anti-Shock Garment (NASG) is a first-aid device to reduce mortality from severe obstetric hemorrhage, the leading cause of maternal mortality globally. We sought to evaluate patient characteristics associated with mortality among a cohort of women treated with the NASG in Nigeria. Data on 1,149 women were collected from 50 facilities participating in the Pathfinder International Continuum of Care: Addressing Postpartum Hemorrhage project in Nigeria from 2007–2012. Characteristics were compared using the appropriate distributional tests, and we estimated multivariable logistic regression models to control for treatment received. There were 201 deaths (17.5%). Women who died were significantly more likely to have any co-morbidity (AOR 3.63, 95% CI: 2.41–5.48), ruptured uterus (AOR 2.79, 95% CI: 1.48–5.28), macerated stillbirth (AOR 2.96, 95% CI 1.60–5.48) and to have had 6 or more previous births, (AOR 1.53, 95% CI 1.11–2.12), after adjusting for treatment received. These results suggest certain maternal conditions, particularly the presence of another life-threatening co-morbidity or macerated stillbirth, conferred a higher risk of mortality from PPH. This underscores the need for multi-system assessment and a comprehensive approach to the treatment of women with pregnancy complications.

## Introduction

Obstetric hemorrhage (OH) is the leading cause of maternal mortality globally, responsible for at least 25% of the 287,000 maternal deaths estimated to occur annually [1], [2]. Most of these deaths occur postpartum, and are due to uterine atony [3]. Other common etiologies of obstetric hemorrhage include complications of abortion, genital tract trauma, uterine rupture, retained placental tissue, and maternal coagulation disorders. Active management of third-stage labor (AMTSL), particularly the administration of prophylactic uterotonics, will prevent 20–60% of atonic postpartum hemorrhage (PPH) [4], [5]. However, not all PPH is due to uterine atony. Immediate treatment is critical to prevent mortality and morbidity due to PPH and OH [6], [7]. Treatment protocols include rapid administration of intravenous crystalloid fluid, uterotonics, bimanual uterine massage, manual removal of the placenta, repair of lacerations, blood transfusion and surgery [8].

**Figure 1:**
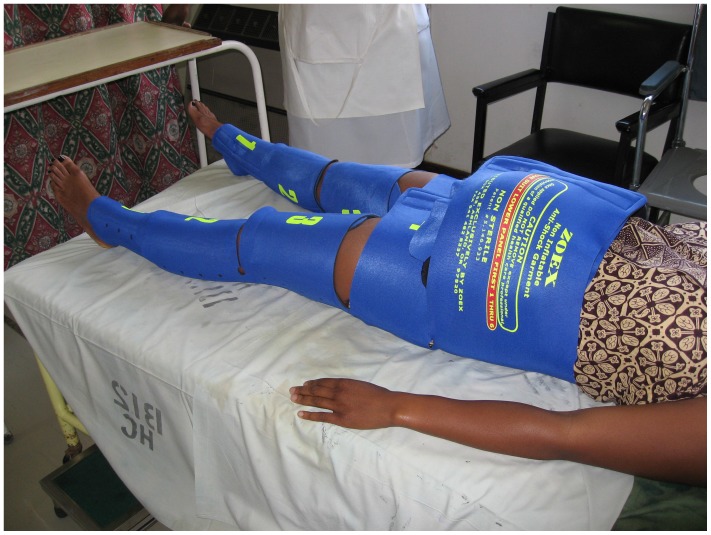
The Non-pneumatic Anti-Shock Garment.

In many low-resource settings, four primary delays contribute to higher rates of maternal morbidity and mortality by increasing the time from onset of the obstetric complication to receipt of care: delay in problem recognition, delay in deciding to seek skilled obstetric care, delay in reaching a facility that can provide treatment, and delay at referral facilities in providing quality emergency treatment [9], [10]. The Non-pneumatic Anti-shock Garment (NASG) is a first-aid compression device that can surmount such delays by stabilizing women in hypovolemic shock secondary to obstetric hemorrhage until definitive treatment can be obtained. The NASG places circumferential pressure on the lower half of the body and compresses the uterine arteries to decrease blood flow to the uterus and reverse shock by increasing circulation to the heart, lung, and brain (Figure 1). NASG intervention as first-aid at the tertiary care level is associated with a nearly 50% reduction in the odds of death from obstetric hemorrhage [11]–[15]; however, it does not constitute definitive treatment. Without access to comprehensive emergency obstetric care (CEmOC), some women will die from hypovolemic shock and multiple organ dysfunction syndromes, even with use of the NASG.

While the severity of shock and hemorrhage are known to modify the risk of mortality among women despite NASG treatment, it is not known whether other maternal characteristics such as communicable and non-communicable comorbidities also affect mortality [16]. Therefore, the purpose of this analysis was to evaluate whether maternal characteristics, including pregnancy and delivery characteristics, etiology and severity of hemorrhage, treatment received, and comorbidities including HIV, malaria, hypertension, anemia, and others were associated with mortality from hypovolemic shock secondary to obstetric hemorrhage despite NASG treatment, within the context of a large-scale postpartum hemorrhage implementation project in Nigeria.

## Methods

### Ethics Statement

These data were obtained from a large-scale community and clinical implementation project conducted by a non-governmental organization with ministerial and institutional collaboration, with the goal of preventing and managing PPH. No consent was necessary from patients for receiving standard of care, which included the NASG at these facilities. Data were collected for evaluation purposes, and case forms did not include personal identifiers. Principal Investigator Dr. Suellen Miller sought human subjects approval from the Committee on Human Research at the University of California, San Francisco (UCSF) but was denied the requested review because the analysis involved the use of de-identified data and thus was not considered human subjects research.

Pathfinder International implemented the *Continuum of Care: Addressing Postpartum Hemorrhage* (CCA-PPH) project in Nigeria from 2007–2012. The project's five-pronged strategy to prevent and manage PPH included training providers in AMTSL; use of a method for accurately measuring blood loss after delivery; obstetric hemorrhage and shock management; use of the NASG to stabilize women in shock secondary to hemorrhage; community mobilization and behavior change communication to encourage antenatal care, birth planning, and timely recognition of emergency situations; and enhanced communication and transportation systems to get women with PPH to the care that they need [17], [18]. Within the CCA-PPH project, Pathfinder implemented the NASG in 60 facilities and 42 communities in seven states (Kano, Katsina, Oyo, Lagos, Nasarawa, Ebonyi, and Yobe). Women with PPH and hypovolemic shock were treated with a standard hypovolemic shock and hemorrhage protocol, in addition to receiving the NASG [19].

Data were collected on women with severe obstetric hemorrhage and shock who were admitted to one of the 60 study facilities between July 2008 and December 2011. Although the project was designed as a PPH project, women with severe obstetric hemorrhage of all etiologies received the NASG if they developed hypovolemic shock, as providers would not reserve the NASG only for women with PPH. Women were treated with the NASG upon presentation with severe obstetric hemorrhage and shock, defined as initial estimated blood loss of >1000 mL and at least one clinical sign of shock (systolic blood pressure <90 mmHg or pulse >110 beats per minute). Providers were also trained to recognize additional signs of shock including pallor, sweating, cold skin, rapid breathing, alterations in consciousness (anxious or confused, unconscious), and oliguria (<30 ml/hr). A total of 1,279 data collection forms (DCFs) were obtained from 50 facilities. Fourteen DCFs were excluded because they were identified as duplicate abstractions on the same case. We further excluded cases where hemorrhage was due to a non-obstetric etiology (n = 4), outcome was unknown (n = 4), patients were referred to a non-study facility (n = 15), patients died from a non-hemorrhage cause (n = 9); and where the NASG was never applied (n = 85). After these exclusions, 1,149 cases remained in our analytic sample.

Data were collected on the following variables: age, gravidity, delivery location, booked status, systolic and diastolic blood pressure, estimated blood loss (mLs), temperature (degrees Celsius), respiratory rates, pulse, hemoglobin, hemorrhage etiology, fetal status at delivery, treatments received, and comorbidities. For study entry, estimates of blood loss were made with a variety of techniques/devices including a calibrated closed-end plastic blood drape, visual estimation, calibrated jugs, and number of soaked clothes/rags. The severity of a woman's shock on study entry was calculated using mean arterial pressure (MAP =  [2*Diastolic Blood Pressure] + Systolic Blood Pressure/3). For analysis purposes, we categorized MAP into <60 mmHg versus ≥60 mmHg, where MAP of 60 was considered the minimum value for adequate oxygen to perfuse tissues [20]. All deliveries occurred at either the health facility, home, or were unknown/unrecorded. Hemorrhage etiologies included uterine atony, complications of abortion, placenta previa, placental abruption, ectopic pregnancy, ruptured uterus, placenta accreta, genital lacerations, retained placenta or fragments, and other. Fetal status at delivery was categorized as alive or dead (fresh still birth or macerated stillbirth). Variables were created to assess the type and amount of treatments received, including IV fluids and blood transfusions. Comorbidities included both communicable and non-communicable disorders: anemia, hypertensive disorders of pregnancy (HDP), sepsis, malaria, HIV/AIDS, and other (convulsions; coagulopathy and pulmonary edema; and history of dizziness, weakness and fainting spells). Hypertensive disorders of pregnancy comprised gestational hypertension, pre-eclampsia and eclampsia.

Clinician data collectors were nurse/midwives or community health workers that were trained onsite in a standardized PPH and shock protocol, collection and measurement of blood loss and completion of data collection forms [18]. Data were collected prospectively during care. Data supervisors cross checked facility records for cases, and where necessary, abstracted cases from the medical records. Paper data forms were reviewed by data supervisors and the Principal Investigator, copied and sent to the University of California, San Francisco where data were entered into a Microsoft Access database (Redmond, WA, USA) and checked for errors and inconsistencies.

Differences between those who survived and those who died were compared using Wilcoxon rank sum test for non-normally distributed continuous variables, and chi-squared or Fisher's exact test for categorical variables. Finally, multivariable logistic regression models were estimated to evaluate factors significantly associated with mortality while controlling for treatment variables using STATA (v 11, College Station, TX). Differences were considered statistically significant at p<0.05.

## Results

Of the study population (n = 1,149), 948 women (82.5%) survived and 201 women (17.5%) died within the hospitalization period. The demographic characteristics, hemorrhage etiologies, fetal outcomes, cause of hemorrhage, condition on study entry and treatments received are presented in Table 1. The proportion of women with high gravidity (≥6 previous pregnancies) was significantly higher for women who died compared to those who survived (49.7% vs. 40.9%, p = 0.026). The distribution of treatment facility type varied significantly by outcome, with a higher proportion of those who died treated in a tertiary care facility (63.7% vs. 52.3%, p = 0.002). We observed a significantly different distribution of hemorrhage etiology by outcome, where higher proportions of women who died had uterine atony (51.8% vs. 46.9% for survived versus died, respectively), placental abruption (11.4% vs. 8.3%), placenta previa (4.7% vs. 3.2%), and ruptured uterus (7.8% vs. 3.7%). There were also significant differences in fetal outcome: 16.2% of women that died had a macerated fetus, vs. only 6.2% of survivors (p<0.001).

**Table 1:** Characteristics of Women with Hypovolemic Shock Secondary to Obstetric Hemorrhage at Time of Study Entry, Nigeria (n = 1,149).

Characteristic	Survived	Died	P
**Number of women**	948 (82.5)	201 (17.5)	
**Age, mean (sd)** δ	29.1 (6.7)	30.5 (6.9)	0.031
**Gravidity, mean (sd) ^φ^**	5.1 (3.4)	5.7 (3.5)	0.032
**High gravidity (≥6) ^φ^**	358 (40.9)	94 (49.7)	0.026
**Booked^€^**	52 (10.7)	10 (11.0)	0.930
**Delivery at Health Facility^£^**	512 (57.7)	99 (63.9)	0.152
**Treatment Facility Type^φ^**			
Primary	101 (10.7)	9 (4.5)	0.002
Secondary	345 (36.4)	61 (30.4)	
Tertiary	496 (52.3)	128 (63.7)	
**Hemorrhage Etiology^¥^**			0.001
Uterine Atony	439 (46.9)	100 (51.8)	
Retained Placenta or Fragments	219 (23.4)	26 (13.5)	
Complications of Abortion	69 (7.4)	7 (3.6)	
Placental Abruption	78 (8.3)	22 (11.4)	
Placenta Previa	30 (3.2)	9 (4.7)	
Ruptured Uterus	35 (3.7)	15 (7.8)	
Lacerations	38 (4.1)	8 (4.2)	
Ectopic Pregnancy	15 (1.6)	0 (0)	
Placenta Accreta	2 (0.2)	0 (0)	
Other	12 (1.3)	6 (3.1)	
**Fetal Outcome^∞^**			0.001
Alive	418 (61.6)	58 (52.3)	
Dead: Fresh still birth	219 (32.3)	35 (32.5)	
Dead: Macerated	42 (6.2)	18 (16.2)	
**MAP<60 mmHg^µ^**	273 (43.8)	43 (41.8)	0.704
**Temperature <37.0 C^α^**	218 (49.3)	44 (60.3)	0.083
**Respiratory Rate, mean (sd)^ β^**	30.1 (13.7)	34.7 (19.3)	0.000
**Pulse, mean (sd)^ π^**	108.4 (21.3)	112.9 (19.1)	0.066
**Estimated blood loss, median (range)^ ρ^**	1000 (100–6000)	1500 (150–4700)	0.001
**IV Fluid received**	847 (89.4)	173 (86.1)	0.181
**Volume IV Fluid, median (range) ^ϑ^**	2000 (300–12700)	2500 (100–7500)	0.058
**Blood Received**	848 (89.5)	141 (70.2)	0.000
**Units of Blood Received, median (range) ^ 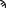 ^**	2 (0.5–12)	1 (0.5–14)	0.000
**Blood Received if MAP<60 mmHg^ 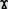 ^**	253 (92.7)	34 (79.1)	0.004
**Comorbidities**			
Any Comorbidity	76 (8.0)	49 (24.4)	0.000
Anemia	30 (3.2)	28 (13.9)	0.000
Hypertensive Disorders of Pregnancy	43 (4.5)	18 (9.0)	0.011
Sepsis	2 (0.2)	5 (2.5)	0.002
Malaria	4 (0.4)	1 (0.5)	1.000
HIV/AIDS	0 (0)	1 (0.5)	0.175
Other	3 (0.3)	0 (0)	1.000

δ N^o^ women survived: 890, N^o^ women died: 183; **^φ^**N^o^ women survived: 875, N^o^ women died: 189; **^€^**N^o^ women survived: 487, N^o^ women died: 91; **^£^**N^o^ women survived: 887, N^o^ women died: 155; ^φ^N^o^ women survived:942, N^o^ women died:198; **^¥^**N^o^ women survived: 937, N^o^ women died: 193; **^∞^** N^o^ women survived: 679, N^o^ women died: 111; **^µ^** N^o^ women survived: 624, N^o^ women died: 103; **^α^** N^o^ women survived: 442, N^o^ women died: 73; **^β^** N^o^ women survived: 596, N^o^ women died: 104; **^π^** N^o^ women survived: 627, N^o^ women died: 104; **^ρ^** N^o^ women survived: 586, N^o^ women died: 109; **^ϑ^** N^o^ women survived: 847, N^o^ women died: 173; **^
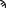
^** N^o^ women survived: 848, N^o^ women died: 141; **^
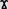
^** N^o^ women survived: 273, N^o^ women died: 43.

Certain characteristics of maternal condition on study entry varied by survival status (Table 1). Women who died had 500 mL higher median estimated blood loss (1500 vs. 1000, p = 0.001), and higher respiratory rate (34.7 vs. 30.1, p<0.001) than women who survived. Comorbidities were significantly more prevalent among women who died (24.4% vs. 8.0%, p<0.001). Specifically, women who died had higher rates of anemia (13.9% vs. 3.2%, p<0.001), HDP (9.0% vs. 4.5%, p = 0.011), and sepsis (2.5% vs. 0.2%, p = 0.002) than those who survived.

Treatments administered during resuscitation from hypovolemic shock are also presented in Table 1. While there was no significant difference in the proportion of women who received IV fluid treatment, women who survived received 20% lower median volume IV fluid than those who died (2000 mL vs. 2500 mL, p = 0.058). The proportion of women who received blood transfusion was significantly lower among those who died (70.2% vs. 89.5%, p<0.001). Furthermore, among those who received blood, the median number of units received was lower among those who died than those who survived (1 unit vs. 2 units, p<0.001). Lower rates of blood transfusion were also observed among women who died when analysis was limited to a subset of women with MAP<60 (79.1% vs. 92.7%, p = 0.004).

Multivariable logistic regression analyses were conducted to evaluate whether the association between characteristics significantly associated with death from OH in bivariate analyses persisted when adjusting for treatment with blood transfusions and IV fluids (Table 2). Compared to survivors, women who died were significantly more likely to have any comorbidity (AOR 3.63, 95% CI: 2.41–5.48). Disaggregated, the individual comorbidities that were associated with significantly higher odds of mortality included anemia (AOR 4.64, 2.65–8.10), HDP (AOR 2.13, 95% CI 1.18–3.85), and sepsis (AOR 9.60, 95% CI 1.73–52.36). Mortality was also significantly associated with ruptured uterus (AOR 2.79, 95% CI 1.48–5.28), macerated stillbirth (AOR 2.96, 95% CI 1.60–5.48) and high gravidity (AOR 1.53, 95% CI 1.11–2.12).

**Table 2:** Odds Ratios for Death by Selected Participant Characteristics, Unadjusted and Adjusted for Treatment Receipt, Nigeria (n = 1,149).

	Unadjusted		Adjusted	
	OR	95% CI	OR	95% CI
**Any comorbidity**	3.70	(2.48, 5.51)	3.63	(2.41, 5.48)
Anemia	4.95	(2.89, 8.50)	4.64	(2.65, 8.10)
HDP	2.07	(1.17, 3.67)	2.13	(1.18, 3.85)
Sepsis	12.07	(2.32, 62.64)	9.60	(1.73, 53.26)
Malaria	1.18	(0.13, 10.61)	1.54	(0.17, 13.89)
**Cause of Hemorrhage: Ruptured Uterus**	2.17	(1.16, 4.06)	2.79	(1.48, 5.28)
**Fetal outcome: Macerated stillbirth**	2.94	(1.62, 5.31)	2.96	(1.60, 5.48)
**High Gravidity (≥6)**	1.43	(1.04, 1.94)	1.53	(1.11, 2.12)
**No Receipt of Blood**	3.61	(2.50, 5.21)	3.66	(2.47, 5.15)
**No Receipt of IV Fluids**	1.36	(0.87, 2.13)	1.21	(0.76, 1.93)

*Note: adjusted analyses controlled for treatment variables: any blood transfusion and any IV fluid receipt.*

## Discussion

These results suggest that certain characteristics or conditions of the woman and of the health care system are associated with the risk of death from obstetric hemorrhage despite application of the NASG. The 201 women who died regardless of NASG treatment were significantly more likely to have a comorbidity, a macerated stillbirth, or ruptured uterus hemorrhage etiology, even when controlling for receipt of blood transfusion and IV fluid. Women who died were also less likely to have received blood transfusion.

We found that the presence of any comorbidity conferred a four-fold increased risk of death. Evaluated individually, sepsis, anemia and HDP were all significantly associated with increased odds of mortality, whereas malaria was not. Of the individual comorbidities, sepsis had the strongest association with mortality, at 9.6 the odds of mortality, followed by anemia at 4.6 and HDP, at 2.1. These conditions are individually associated with increased risk of maternal death; however, whether an additive or multiplicative interaction effect occurs when in conjunction with hemorrhage is not evident from the literature. Sepsis is considered a primary contributor to maternal death, particularly in low-income countries, and is estimated to be responsible for 9.7% of maternal deaths in Africa [2], [21]. Anemia has been found to contribute to maternal death for both acute onset and chronic conditions, including cardiac failure, susceptibility to infection, and more severe postpartum blood loss [2], [22]–[25]. Malaria is another contributor to anemia in pregnant women; however, the prevalence of malaria infection in our sample was relatively low and did not vary across maternal outcome. Previous to 2012, providers had questioned whether pre-eclamptic/eclamptic patients might face an increased risk of cerebral intracranial hemorrhage with NASG intervention, which increases blood flow to the heart, lung, and brain; however, an analysis by Ismail, *et al.* indicated that the NASG intervention among pre-eclamptic and eclamptic patients actually reduced the odds of an extreme adverse outcome by 74% (OR 0.26, 95% CI 0.07–0.93) [26]. Results from the current analysis suggest that women with HDP who receive the NASG may be more at risk of dying of conditions related to their hypertension or eclamptic sequelae, such as disseminated intravascular coagulation (DIC) or HELLP syndrome, than conditions related to their hemorrhage. The finding that women suffering from any comorbidity, and in particular, from sepsis, anemia and HDP, have a greater risk of death than those without, emphasizes the need for clinicians to conduct multi-systems assessments of women seeking emergency care for obstetric hemorrhage, so that they can effectively manage all conditions that may influence her survival.

One unexpected finding was that malaria and HIV infection were not found to be associated with increased risk of death for women with obstetric hemorrhage. Other studies have found four and five-fold increases in maternal death rates among HIV-infected women [27], [28]. One possible explanation is that HIV/AIDS and malaria were under-reported in this data. The median prevalence of HIV/AIDS among pregnant women in Nigeria is 4.1%, and ranges regionally to at least 8.2% [29], [30], compared to our data where the prevalence is 0.1%. One study suggested the prevalence of malaria to be 7.7% among pregnant women in Lagos [31] compared to 0.5% reported in our data. It is possible that had our dataset been representative of national rates of HIV/AIDS and malaria, we might have seen an association between mortality and these comorbidities.

Our analysis found that women who delivered a macerated stillbirth were three times as likely to die as women with a different fetal outcome (i.e., live birth, fresh stillbirth). The association of life-threatening hemorrhage and DIC with a dead fetus was recognized as early as 1901, when DeLee reported “temporary hemophilia” in a woman with a macerated fetus [32]. Recent hematology studies on women with intrauterine fetal demise have noted elevated fibrin degradation products, believed to be mediated by thromboplastin from the macerated fetus [33]. In a study of macerated stillbirths, Habek noted that potential maternal complications induced by autolytic lesions include DIC, uterine atony, and postpartum hemorrhage [34]. This finding supports efforts to encourage a rapid delivery among women identified to be carrying a dead fetus.

We also found that women with hemorrhage etiology of ruptured uterus were nearly three times as likely to have died as women with another hemorrhage etiology, a result that is consistent with the literature. Uterine rupture typically results in significant intraperitoneal bleeding, requires large amounts of IV fluids and blood, and treatment requires surgical intervention to repair the tear or for a complete hysterectomy. While uterine rupture is uncommon, research suggests that the incidence is higher in areas with higher than average incidence of neglected and obstructed labor due to inadequate access to medical care (0.11%) [35].

Women who did not receive blood transfusion were nearly four times more likely to die than those that did, despite use of the NASG. This finding is consistent with the fact that the NASG is not a replacement for treatment; it is a first-aid device intended to stabilize women with hypovolemic shock until they receive definitive treatment. It may buy the woman more time to enable her to survive longer while waiting for a blood transfusion, but blood replacement may still be necessary. In many low-resource settings, as in Nigeria, such therapy may not be available at all health care facilities, particularly at the primary and secondary level. The lack of universal receipt of blood transfusion (86.1% overall), and the low volume transfused (median 2 units) among those that received blood might be attributable to poor blood availability at health care facilities. Many factors are responsible for this problem, including gross underfunding of the National Blood Transfusion Service of Nigeria, persistent power shortages, and misconceptions among potential blood donors. Private initiatives have begun to improve the availability of blood in some areas, such as Cloverleaf's funding of solar banks to secure and provide emergency blood for obstetric emergencies [36], but large scaling up of such projects in combination with broader structural investment is required to overcome a dire situation.

Prior studies of the NASG at the tertiary care facility level have indicated that greater severity of condition at study entry, described by MAP, was associated with greater risk of death [16], [37], [38]. We did not observe the same relationship in the current analysis; however, the blood pressure variables used to calculate MAP were not consistently reported thus impeding our ability to fully explore this particular relationship.

Strengths of this analysis include the large sample of women within similar care contexts who received the NASG for treatment of hypovolemic shock secondary to obstetric hemorrhage and the use of the NASG in real world situations of busy, understaffed facilities. However, there are several important limitations to consider while interpreting these findings. First, these data are not the result of a randomized clinical trial, but are observational. Second, the methods for data collection were not carried out as initially planned. Data collection forms were to have been filled in concurrently with patient care or immediately after resuscitation and updated in real-time until patients were discharged. Due to demands on the time of health providers, forms were often completed retrospectively, after the patient had been treated or even after discharge. In the worst cases, data collection forms were completed based on medical record abstraction up to three months after the patient's discharge. Included in this process is a dependence on provider report of comorbidity; the implementation project did not prespecify the analysis that we conducted. Data were recorded for monitoring and evaluation purposes only, thus there is a possibility that comorbidities may be under- or over-reported within this sample and lacked important detail, such as the type of sepsis. Third, data on dates and times of NASG application and removal were not always complete; less than two hours of NASG treatment is considered to be inadequate for resuscitation, however we were not able to calculate the duration of NASG treatment for some women in our dataset and therefore unable to determine if the women who died despite receiving NASG had actually received adequate NASG resuscitation. Fourth, data from only 50 of the 60 participating facilities were available for analysis; the 10 facilities that did not contribute forms were low-volume primary health care facilities which likely saw fewer cases of obstetric hemorrhage and referred earlier due to more limited capacity for treatment. The majority of the forms came from only three of the seven states (Kano, Katsina and Oyo); these three states were also all involved in prior research and thus had greater experience with the NASG which may mean that they had more completely integrated it into their standard of care. Finally, there are high proportions of data missing for several characteristics, which precluded our ability to investigate those predictors. It is also important to note that we continued with an analysis of fetal outcome and death despite a high rate of missing data on fetal outcomes, particularly among women who died.

## Conclusions

Our results suggest that while the NASG contributed to the survival of the majority of women suffering from hypovolemic shock secondary to obstetric hemorrhage within a continuum of care for PPH implementation project, efficacy was negatively affected by certain maternal conditions, particularly the presence of another life-threatening comorbidity or carrying a dead fetus. This underscores the need for clinicians treating women suffering from hypovolemic shock secondary to obstetric hemorrhage to conduct a multi-systems assessment to identify and treat any comorbidities that may adversely affect a woman's pregnancy outcome even if she receives life-saving interventions for hemorrhagic shock. Furthermore, while NASG intervention may mitigate blood loss after application, and buy time during delays in obtaining a transfusion, it does not replace the need for rapid and adequate blood and fluid replacement.

Reducing maternal mortality from obstetric hemorrhage and hypovolemic shock requires timely treatment with IV fluids, adequate blood replacement, and identification and treatment of comorbidities. The NASG is a first-aid device that helps women with hemorrhage survive transport and delays in accessing quality definitive treatment; however, the efficacy of the NASG is enhanced when used in a functioning health system prepared to manage all maternal complications. More investments are needed to strengthen the health system generally to be able to provide quality emergency obstetric care.
